# Obstructive Jaundice Associated with Polcystic Liver Disease

**DOI:** 10.1155/1996/83547

**Published:** 1996

**Authors:** J. Dmitrewski, S. Olliff, J. A. C. Buckels

**Affiliations:** 1 Liver Unit Queen Elizabeth Hospital Edgbaston Birmingham B15 2TH UK; 2 Department of Radiology Queen Elizabeth Hospital Edgbaston Birmingham B15 2TH UK

## Abstract

A 65 year old patient with polycystic liver disease presented with obstructive jaundice thought to be a cholangiocarcinoma. Subsequent investigations demonstrated a large cyst compressing the confluence of the hepatic ducts. Percutaneous decompression of the biliary tree led to a complication necessitating surgery. Treatment options for symptomatic polycystic liver disease are reviewed.


*HPB Surgery*, 1996, Vol.10, pp. 117–120
Reprints available directly from the publisher
Photocopying permitted by license only

© 1996 OPA (Overseas Publishers Association)
Amsterdam B.V. Published in The Netherlands
by Harwood Academic Publishers
Printed in Malaysia

### CASE REPORT
### Obstructive Jaundice Associated with Polycystic Liver Disease

J. DMITREWSKI, S OLLIFF* and J. A. C. BUCKELS

Liver Unit and *Department of Radiology Queen Elizabeth Hospital Edgbaston,
Birmingham B15 2TH, UK

*(Received 20 July 1994)*

A 65 year old patient with polycystic liver disease presented with obstructive jaundice thought to be a cholangiocarcinoma. Subsequent investigations demonstrated a large cyst compressing the confluence of the hepatic ducts. Percutaneous decompression of the biliary tree led to a complication necessitating surgery. Treatment options for symptomatic polycystic liver disease are reviewed.

KEY WORDS: Obstructive jaundice polycystic liver disease

### INTRODUCTION

Polycystic liver disease is usually asymptomatic. We report a rare case of obstructive jaundice caused by compression of the common hepatic duct by a large infected liver cyst. Specific diagnostic and management problems of this case are presented and the treatment of polycystic liver disease discussed.

### CASE REPORT

A 65 year old male with chronic renal failure due to adult polycystic kidney disease was admitted to his dialysis unit with rapidly progressing jaundice following two months history of abdominal discomfort, nausea and weight loss. Liver function tests showed a picture of obstructive jaundice with bilirubin of 190 µmol/L, alkaline phosphatase 1049 u/L and SGPT 41 u/L. Hepatitis serology was negative.

An US scan was non-conclusive apart from showing the distorted anatomy of large polycystic kidneys and liver. Percutaneous transhepatic cholangiogram revealed a narrowing at the confluence of hepatic ducts thought to be a cholangiocarcinoma (Fig. 1). A pig-tail catheter drain was placed in the biliary tree and the patient was referred to our unit for further assessment. CT scan demonstrated a number of cysts in the liver with a significant amount of normal residual parenchyma. There was one large thickwalled cyst extending from segment IV compressing the confluence of the hepatic ducts and gall bladder (Fig. 2).

Pus was drained from the cyst with a pig-tail catheter and subsequently liver function parameters gradually improved. After two weeks of continuous drainage the jaundice relapsed accompanied by a drop in haemoglobin from 9.6 to 5.4 g/dl over 24 hours. Cholangiogram through the percutaneous catheter revealed extensive haemobilia. Close to the catheter a structure was seen which filled with contrast and then appeared to wash out suggesting a communication with blood vessel. Hepatic angiogram showed a false aneurysm of the left hepatic artery (LHA), immediately adjacent to the catheter placed in the hepatic duct (Fig. 3). The LHA damage was assumed to have been cause either during introduction of the catheter through the liver tissue or by subsequent erosion of the wall of the duct and artery. Selective catheterisation for embolisation of the LHA was not achieved because adequate access via the left gastric artery could not be obtained. Laparotomy was undertaken to control the bleeding and to decompress the biliary tree. The large cyst from segment IV was fenes-

*Correspondence to*: Mr J. A. C. Buckels, Liver Unit, Queen Elizabeth Hospital. Edgbaston. Birmingham B15 2TH

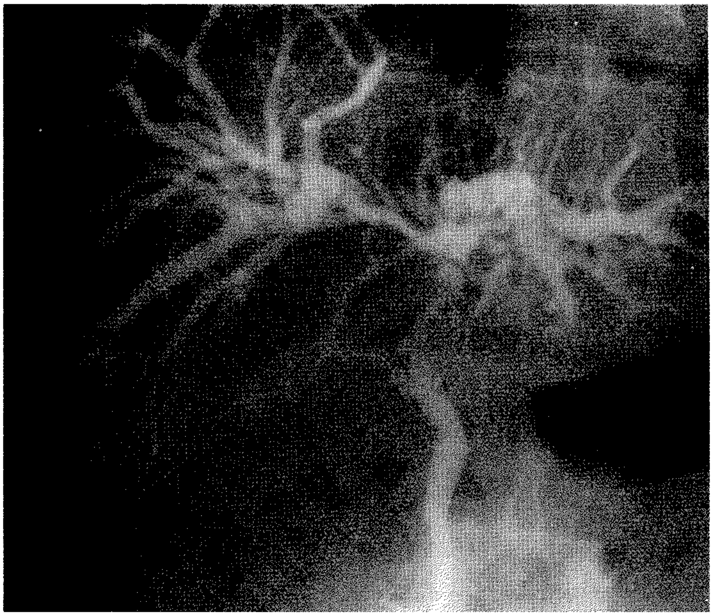

**Figure 1** Percutaneous cholangiogram showing narrowing at the confluence of the hepatic ducts.

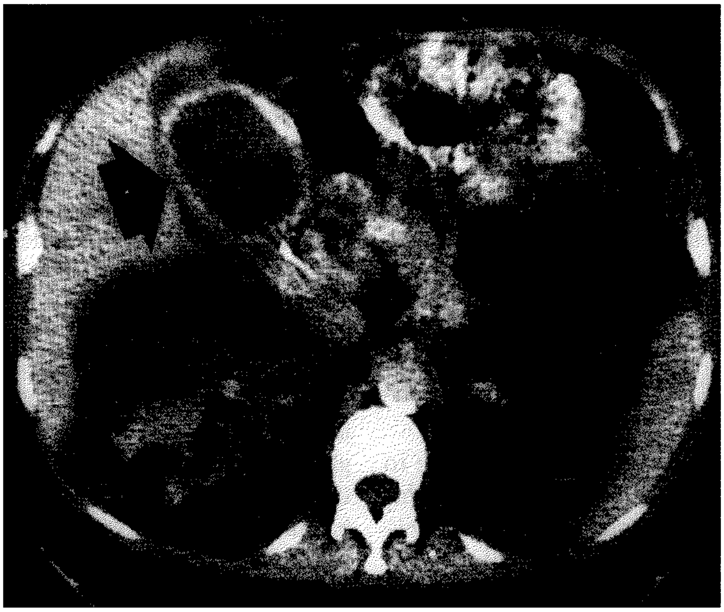

**Figure 2** Gall bladder (arrow) compressed from the left by a large cyst extending from segment IV.

trated and the common hepatic duct opened to remove the debris. Because of the inflammatory changes in the porta hepatis and possibility of late stricture formation formal biliary reconstruction using Roux-en-Y hepaticojejunostomy was performed. The LHA was embolised with collagen and ligated. Post-operatively the patient made a steady recovery with no further bleeding and the jaundice gradually resolving. He remains well with normal liver function 18 months later.

### DISCUSSION

Polycystic liver disease (PLD) is a rare disorder of liver parenchyma characterised by the presence of multiple

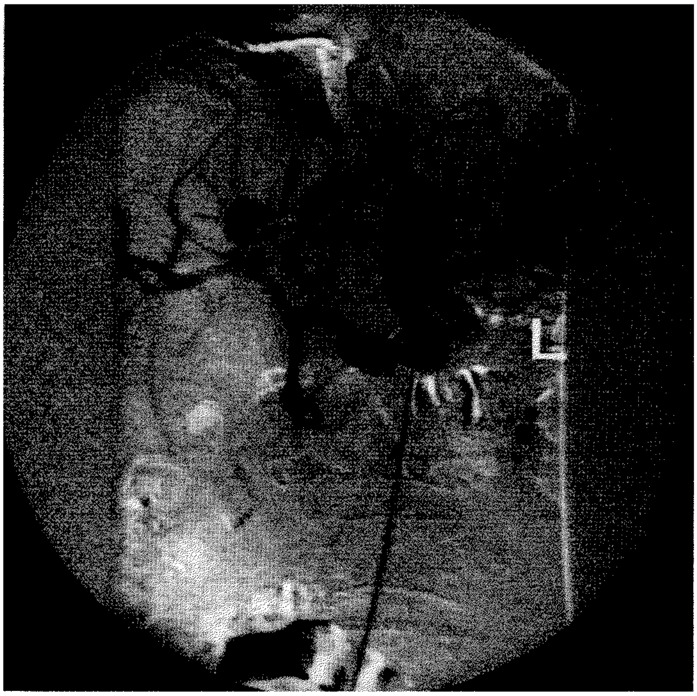

**Figure 3** False aneurysm of the left hepatic artery. Percutaneous drainage catheter immediately adjacent to the aneurysm.

cystic lesion. It usually accompanies adult polycystic kidney disease, an autosomal dominant abnormality. Uncommon in general population with an autopsy prevalence of 0.13%[1], the incidence of PLD among patients with chronic renal failure due to polycystic kidneys exceeds 50%[2].

The commonest symptoms in patients with PLD are related to increase in liver size and include abdominal discomfort and respiratory compromise. Liver dysfunction is generally rare. The liver parenchyma is usually well preserved even in cases with massive cysts and this is reflected by normal liver function tests found in majority of PLD patients[3]. However it has been shown that in patients with chronic renal failure due to PKD and coexisting liver involvement hepatic complications account for a significant morbidity and mortality[4]. As the liver cysts progress slower than the renal disease such complications are more likely to affect patients benefiting from long-term dialysis or kidney transplantation. Cyst infection and malignant transformation within a cyst have been reported[4]. Jaundice is an unusual feature of PLD and to our knowledge obstructive jaundice due to compression of the extrahepatic biliary tree by a liver cyst has not been previously documented.

The treatment options of symptomatic PLD are largely percutaneous drainage or open surgical repair. Percutaneous aspiration and drainage carry high recurrence rate though sclerotherapy using 95% ethanol may produce prolonged symptomatic improvement in selected patients with a single or small number of cysts.

As seen in our case percutaneous techniques are not free from risk. Vascular injuries are recognised consequence of percutaneous biliary drainage but are rarely symptomatic.

Surgical treatment remains controversial and its long-term benefit has not yet been proven. To date cyst fenestration remains the most favoured option. Recent reports suggest fenestration combined with segmental liver resection in patients with clearly defined cyst complexes[5]. In those rare cases in which symptoms led to end-stage disability liver transplantation has been advocated. So far however the experience remains limited[6].

### REFERENCES

1. Kwok M. K, Lewin K. J. (1988) Massive hepatomegly in adult polycystic liver disease. *Am J Surg Pathol* **12**: 321–24.
2. Thomsen H. S, Thaysen J. H. (1988) Frequency of hepatic cysts in adult polycystic kidney disease. *Act Med Scand* **224**: 381–84.
3. Everson G. T, Scherzinger A, Berger-Leff N *et al.* (1988) Polycystic liver disease: quantitation of parenchymal and cysts volumes from computed tomography images and clinical correlates of hepatic cysts. *Hepatology* **8**: 1627–34.
4. Grunfeld J. P, Albouze G, Jungers P *et al.* (1984) Liver changes and complications in adult polycystic kidney disease. *Adv Nephrol* **14**: 1–20.
5. Newman K. D, Torres V. E, Rakela J, Nagorney D. M. (1990) Treatment of highly symptomatic polycystic liver disease. *Ann Surg* **212**: 30–37.
6. Starzl T. E, Reyes J, Tzakis A, Mieles L, Todo S, Gordon R. (1990) Liver transplantation for polycystic liver disease. *Arch Surg* **125**: 575–77.

